# Dissociable functional activities of cortical theta and beta oscillations in the lateral prefrontal cortex during intertemporal choice

**DOI:** 10.1038/s41598-018-21150-1

**Published:** 2018-07-25

**Authors:** Dan-Yang Gui, Tao Yu, Zhenhong Hu, Jiaqing Yan, Xiaoli Li

**Affiliations:** 1 Department of Marketing, College of Management, Shenzhen University, Shenzhen, China; 2 State Key Laboratory of Cognitive Neuroscience and Learning & IDG/McGovern Institute for Brain Research, Beijing Normal University, Beijing, China; 3 Beijing Institute of Functional Neurosurgery, Xuanwu Hospital, Capital Medical University, Beijing, China; 4 J. Crayton Pruitt Family Department of Biomedical Engineering, University of Florida, Gainesville, FL 32611-6131 USA; 5 College of Electrical and Control Engineering, North China University of Technology, Beijing, China

**Keywords:** Decision, Neurophysiology

## Abstract

The lateral prefrontal cortex (LPFC) plays an important role in the neural networks involved in intertemporal choice. However, little is known about how the neural oscillation of LPFC functions during intertemporal choice, owing to the technical limitations of functional magnetic resonance imaging and event-related brain potential recordings. Electrocorticography (ECoG) is a novel neuroimaging technique that has high spatial and temporal resolution. In this study, we used ECoG and projected the ECoG data onto individual brain spaces to investigate human intracranial cortex activity and how neural oscillations of the LPFC impact intertemporal choice. We found that neural activity of theta oscillation was significantly higher during impulsive decisions, while beta oscillation activity was significantly higher during non-impulsive ones. Our findings suggest a functional dissociation between cortical theta and beta oscillations during decision-making processes involved in intertemporal choice, and that decision outcomes may be determined by LPFC modulation, which involves neural oscillations at different frequencies.

## Introduction

Humans and animals are often unwilling to wait long for delayed rewards^1,2^. One would thus rather choose a smaller and immediate reward instead of waiting for a larger and later reward. Many choices in our lives are intertemporal and involve tradeoffs between short-term outcomes and long-term benefits. For instance, children may prefer to play video games rather than do homework, adults may choose to spend their salary to buy a car rather than making a long-term investment in the bank, or eat junk food rather than healthy meals that require longer preparation times. Intertemporal choice is a classic economic decision-making task. It refers to decision-making regarding outcomes (reward or loss) that occur at different time points^3,4^. Previous studies have shown that individuals tend to discount future reward in intertemporal choice. However, there are tremendous individual differences in the time discounting rate, which correlates with behaviors of real-life circumstances and clinical disorders involving aself-control. These disorders include gambling and addiction^5–7^.

Neuroscientists aim to identify the specific neural mechanisms underlying decision-making during intertemporal choices. Previous studies using functional magnetic resonance imaging (fMRI) and electrophysiology have shown that the ventral striatum (VS), ventromedial prefrontal cortex (vmPFC), and posterior cingulate cortex (PCC) represent the subjective valuation of available decision options^8–12^. The prefrontal cortex and lateral parietal cortex have been associated with self-control and choice processes^13–15^. Previous event-related brain potential (ERP) studies on intertemporal choice have found that the P200 and P300 components vary as the temporal distance for the reward increases from 2 weeks to 50 years^16^. There is also evidence that top-down attention filtering occurs early during the decision period, and that value modulation occurs later in the process (450–650 ms after stimulus onset)^17^. Our previous studies have revealed that the P200 component might reflect an initial valuation of reward and a time delay, while the frontal N2 component correlates with individual preference for immediate reward^18^; more impulsive individuals exhibit larger P200 components^19,20^.

The LPFC plays an important role in the neural networks involved in intertemporal choice. Previous studies have shown that the neural activity of LPFC modulates valuation processes and later cognitive components of decision-making^17,21^. However, little is known about the relationship between neural oscillations and intertemporal choice.

Although spatial neural networks and temporal dynamics have been studied extensively using fMRI and electroencephalography (EEG)/ERP, neither fMRI nor EEG/ERP can simultaneously achieve high spatial and temporal resolution. Electrocorticography (ECoG) is a novel neuroimaging technique that has higher spatial (compared to EEG/ERP) and temporal (compared to fMRI) resolution and is used to investigate human intracranial cortex activity^22,23^.

We used ECoG to investigate human intracranial cortex activity, and how neural oscillations of the LPFC impact intertemporal choice. ECoG recordings have several benefits: (1) higher spatial resolution than scalp EEG^22^, which allows us to locate the LPFC accurately; (2) better temporal resolution than fMRI; (3) fewer artifacts from muscles or head movements compared to scalp EEG^22,24^; (4) and a better signal-to-noise ratio compared to scalp EEG and magnetoencephalography (MEG)^25^. Previous studies have suggested that low-frequency oscillations associated with long-range communication between different brain regions^26,27^ and beta oscillation is related to behavioral inhibition^28,29^. Therefore, we expected that neural activities of theta and beta oscillations in the LPFC would be the crucial factors associated with decision outcomes during intertemporal choice.

## Materials and Methods

### Participants

We studied seven adult patients. The following six inclusion criteria were required for participation: (i) aged 18 years or older; (ii) two-stage epilepsy surgery using chronic subdural ECoG recording at the Xuan Wu hospital in Beijing; (iii) implantation of subdural electrodes for epilepsy surgery over the LPFC; (iv) normal listening, speaking, memory retention and mathematical computation abilities (every potential participant performed a short ability test before the experiment) (one patient was excluded); (v) no seizures either within 2 hours prior to the experiment or during the task period of experiment (two patients were excluded); and (vi) brain areas in the LPFC were not involved in seizure onset or subsequent surgical resection (two patients were excluded). A total of five participants were excluded according to our criteria. Informed consent was obtained from each participant during the enrollment. The study protocol was approved by the local ethics committee of Beijing Normal University. All methods were performed in accordance with the approved protocol.

### Task

**Figure 1:**
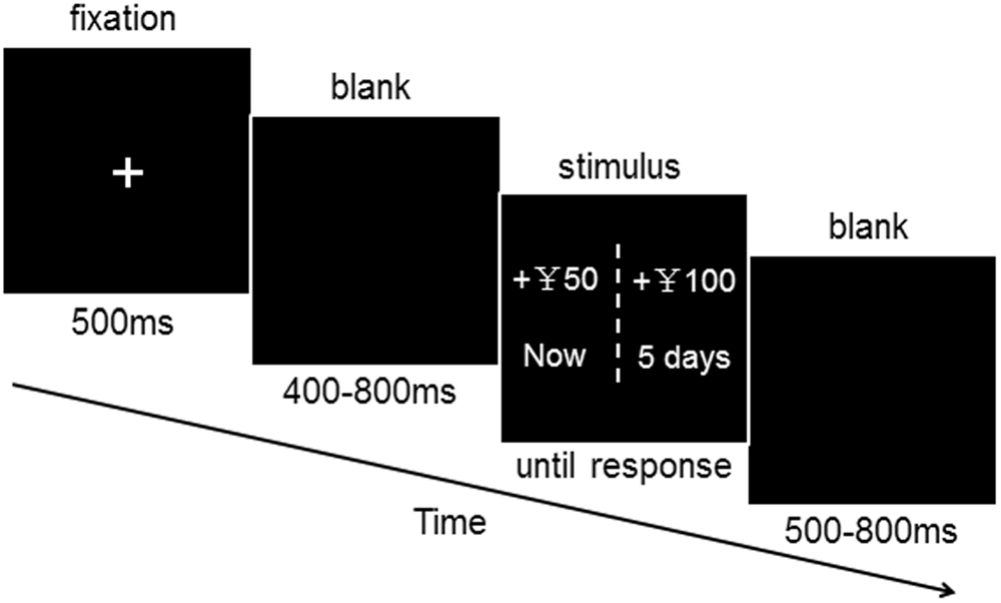
Time course of a single task trial. Each trial began with a 500-ms fixation point and was followed by the blank screen randomized to last between 400 and 800 ms. A screen displaying the stimulus was then shown until the participants responded. The inter-trial interval was randomized to last between 500 and 800 ms.

The procedure of the task is presented in Fig. 1. The entire experiment comprised 80 test and 8 practice trials. The participants were instructed to choose between two monetary-gain alternatives: an immediate and smaller reward (IS) or a later and larger reward (LL). These alternatives were to be obtained at different times (e.g., now vs. 5 days later). For each set of intertemporal alternatives, the IS-money reward was fixed at ¥ 50, and the delayed-money reward was randomly chosen from a predetermined series of monetary amounts ranging from a 20% to 100% increase compared to the IS. The later time points were randomly selected from a predetermined series of delayed periods (one day, three days, or five days later). The two alternatives for each choice were presented on either side of the screen. The locations of the immediate and delayed options were randomly assigned (left or right) on each trial and were counterbalanced across trials. The participants were instructed to press the “F” key to denote a left-sided choice or the “J” key to denote a right-sided choice. The participants were informed prior to the task that they would receive actual payments based on their choices. One of the choices made by the participants was selected at random to determine his or her payoff. If the randomly selected choice was an immediate reward, the participant was paid in cash at the end of the experiment. If the randomly selected option were a delayed reward, the participant would receive the monetary reward at a later date determined by the amount of delay specified in the option. All procedures were performed in accordance with the approved protocol.

### ECoG data acquisition

Platinum electrodes (inter-contact distance: 10 mm; diameter: 4 mm) were surgically placed in the subdural space over cortical regions of the left or right hemisphere. All electrode plates were stitched to adjacent plates or to the edge of the dura mater to prevent the movement of subdural electrodes after intracranial implantation. ECoG recordings were obtained from 64 electrodes using a Brain Product amplifier (Brain Product, GmbH; Germany). The signals were amplified using a 0.01–500-Hz band-pass and continuously sampled at 2,500 Hz in each channel for offline analysis.

### Coregistration of electrodes with individual three-dimensional MRIs

High-resolution T1-weighted MRI scans were acquired before the electrode implantation surgery. Post-implant radiographs (X-rays) were acquired with the subdural electrodes in place to localize the electrodes on the brain surface. Digital photographs were also obtained during surgical implantation of the electrode array. First, we used planar X-ray images and the “Location on Cortex” (LOC) package^30^ to identify the stereotactic coordinates of each grid of electrodes. We reconstructed the three-dimensional brain surface from high-resolution MRIs using SPM8 (Wellcome Trust, see http://www.wellcome.ac.uk/) software. We then projected each patient’s electrode locations onto his or her three-dimensional brain surface using the MATLAB^®^ (R2011a; Natick, MA, USA) program. Finally, we verified the locations of ECoG electrodes by comparing them to the photographs obtained during surgery. Thus, we simultaneously visualized the electrophysiological recordings, the cortex, and the subdural electrodes.

### Data analysis

During offline analyses, the ECoG data were resampled to 1,000 Hz and re-referenced to the average potential of electrodes included in the analysis for each subject individually. Electrodes implanted on cortical tissue that was surgically resected later, were excluded from the analyses. All ECoG data were analyzed in MATLAB^®^ using custom scripts and in SPSS^®^ (Rel. 13; SPSS Inc.; Chicago, IL, USA).

ECoG data obtained from 1,000 ms before to 1,200 ms after stimulus onset (2,200 ms in total) were defined as an epoch for each stimulus. Epochs affected by artifacts derived from prominent body movements or electrographic seizures were excluded from the analyses. We chose the post-stimulus time window according to previous EEG/ERP studies, which found that P200, N200, P300 and 450–650 components were related to intertemporal choice^16–20^. Next, we established two types of time windows: early time window (from post-stimulus 200 ms to 400 ms) and later time window (from post-stimulus 400 ms to 1000 ms) in our study.

For each channel, time-frequency analysis of spectral changes in oscillatory activity were analyzed using a wavelet transform, which provided a good compromise between time and frequency resolution^31^. Time-frequency analysis was performed for each channel by convolving the data using a complex Morlet wavelet *w* (*t, f*_0_) with a Gaussian shape in time (*σ*_*t*_) and frequency (*σ*_*f*_) around the center frequency (*f*_0_). At each time-frequency point, we analyzed the percentage change in power of the time-frequency spectrum (averaged across trials) relative to the mean power at baseline period (−200 ms to onset of task stimulus). Thus, the power was baseline-corrected for each frequency to obtain the relative power change: *Power*(*t, f*)_corrected_ = 100 × (*Power*(*t, f*) − *Power*_*baseline*_(*f*))/Power_baseline_(*f*).

Functional mapping was performed using a linearly superimposed Gaussians interpolation with a standard deviation of 8 mm. The superposition of weighted Gaussian kernels at each electrode locus presents continuous spatial distributions of ECoG power changes (for more details please refer to Miller, *et al*.^30^).

To test the statistical significance for each time-frequency (TF) matrix, an uncorrected *p* value for each TF point was obtained using a bootstrapping approach^32^. We next performed a false discovery rate (FDR) correction for multiple testing. The corrected significance level α was set at 0.05. We used paired *t* tests for mean powers of all trials in the channels of LPFC (region of interest, ROI), to examine the significant changes in oscillatory activity between impulsive and non-impulsive decisions of each participant.

### Data availability statement

The datasets generated and/or analyzed during the current study are available from the corresponding author upon reasonable request.

## Results

**Figure 2:**
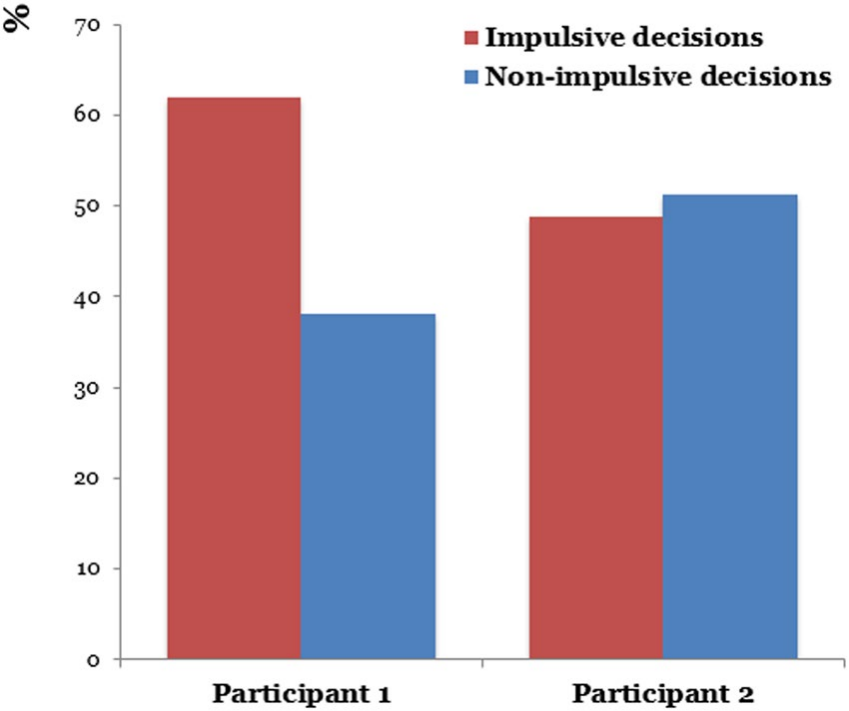
The percentages of impulsive and non-impulsive decisions by the participants.

**Figure 3:**
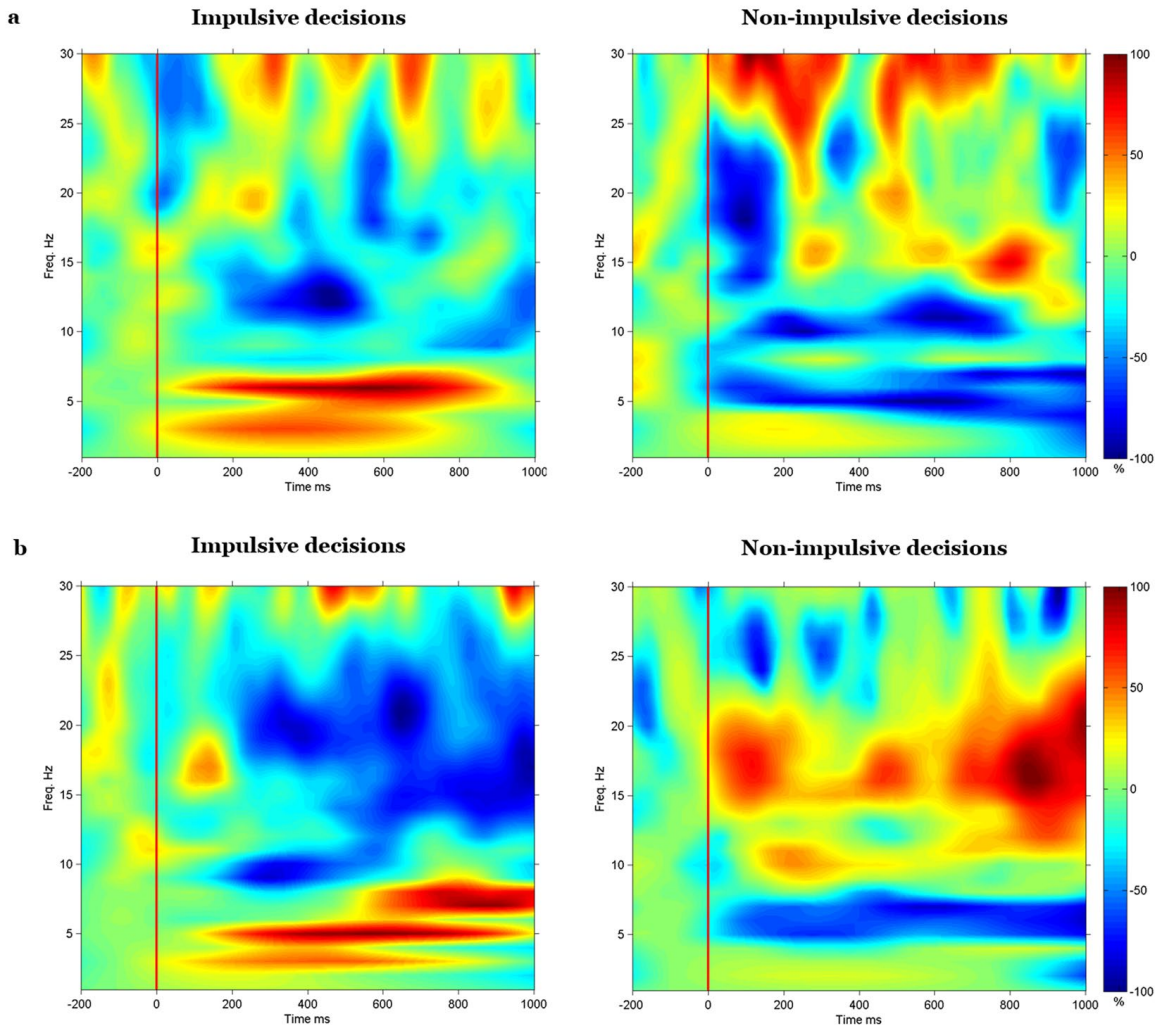
Time-frequency mapping of relative power changes (uncorrected) for one typical left-LPFC channel during the impulsive and non-impulsive decisions of participant 1 (**a**) and participant 2 (**b**). Yellow-to-red colors denote increased relative power (from 0.1% to 100%), while cyan-to-blue colors denote decreased relative power (from −0.1% to −100%). Green color denotes no power change.

Figure 2 shows the impulsive and non-impulsive decisions of two participants during the task. Participant 1 chose more immediate rewards, and participant 2 chose more delayed rewards. Time-frequency analysis of spectral changes in oscillatory activity (Fig. 3) indicated an increased theta oscillatory activity during the impulsive decision and increased beta oscillatory activity during the non-impulsive decision. The mean response time (RT) of participant 1 was 2578.56 ms (from 1318 to 3133 ms) during the impulsive decision and 1444.38 (from 519 to 2422 ms) during the non-impulsive decision; that of participant 2 was 2322.65 ms (from 1391 to 4387 ms) during the impulsive decision and 2113.59 ms (from to 1093 to 3982 ms) during the non-impulsive decision.

**Figure 4:**
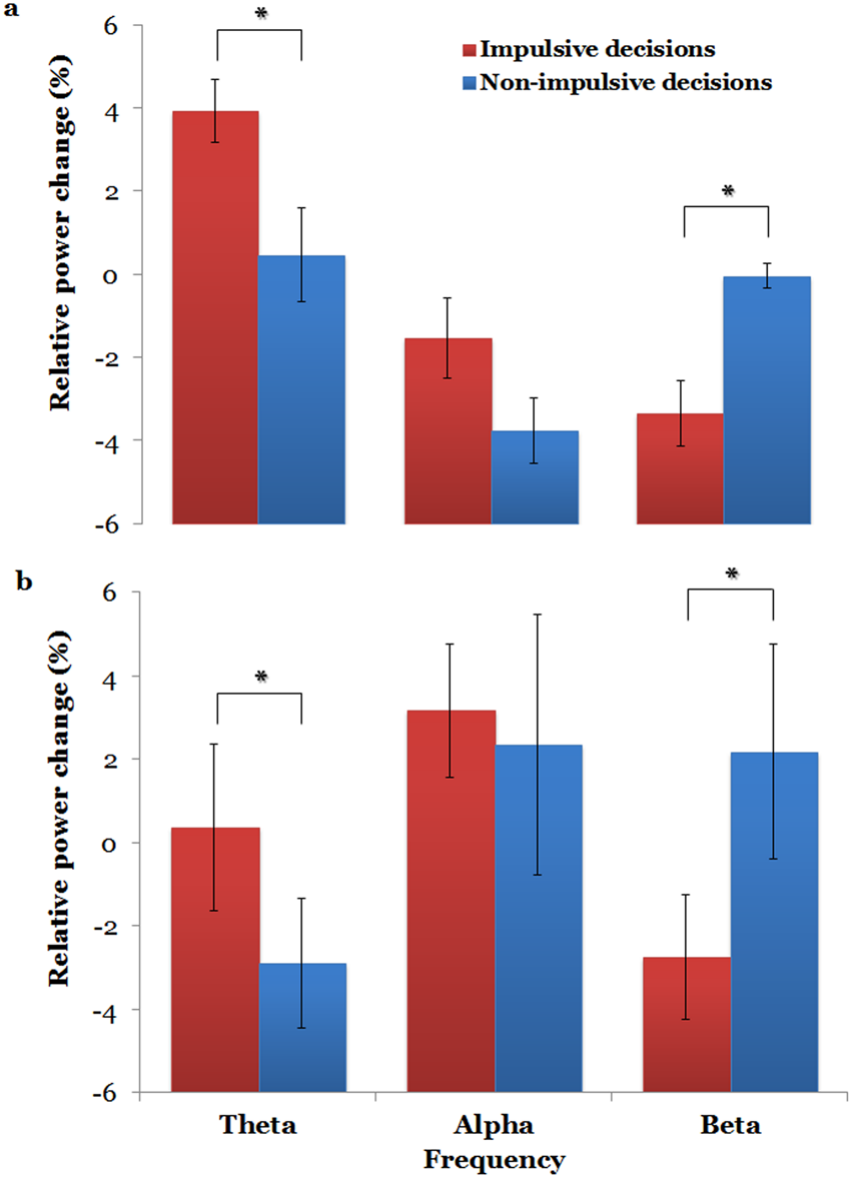
Average relative power changes in all left-LPFC channels in theta, alpha, and beta oscillations during the impulsive and non-impulsive decisions of participant 1 (**a**) and participant 2 (**b**) from post-stimulus 400 ms to 1000 ms. Error bars denote standard error of the mean. *p < 0.05.

**Figure 5:**
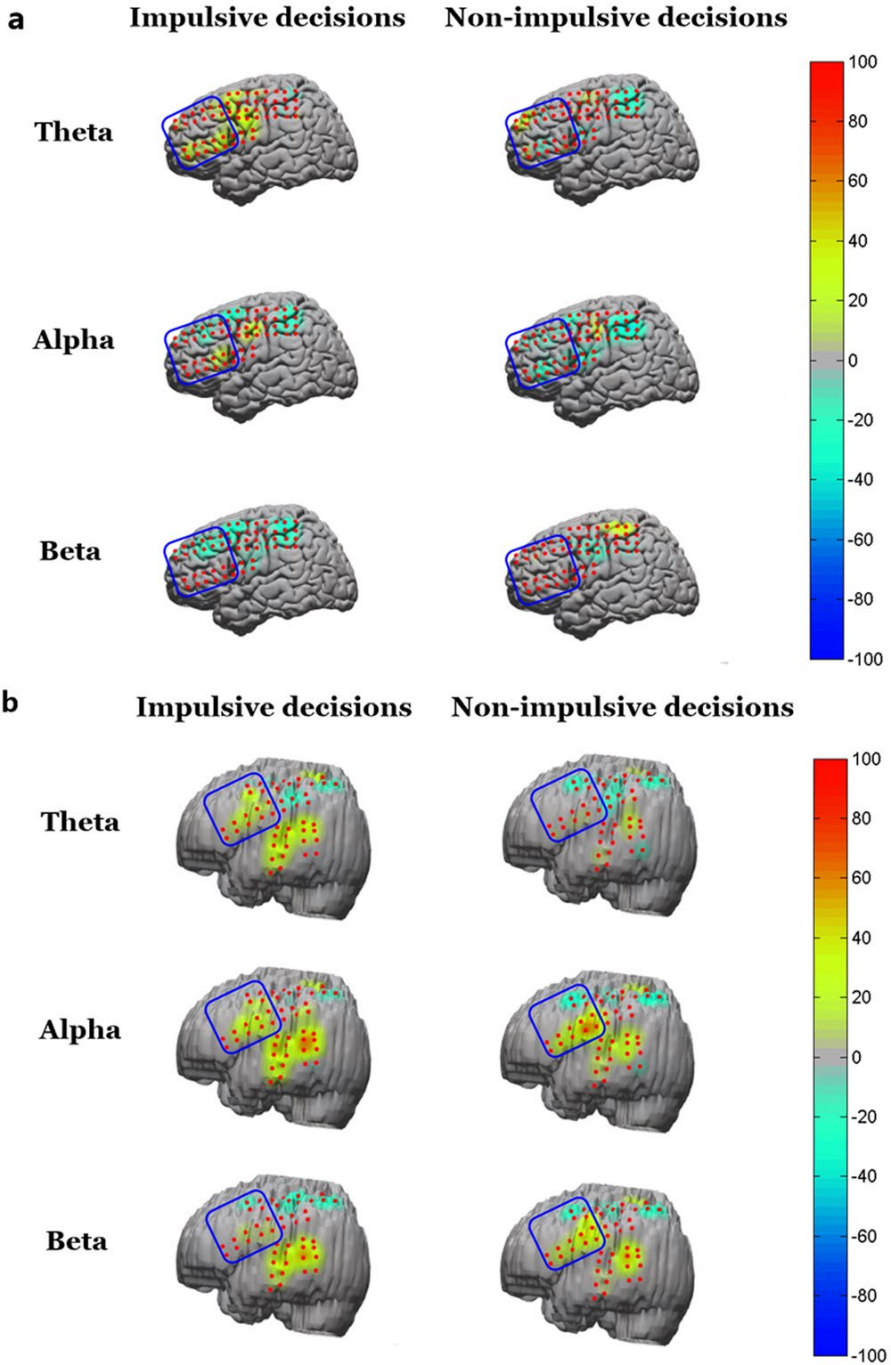
Brain mapping of relative power changes in theta, alpha, and beta oscillations during impulsive and non-impulsive decisions of participant 1 (**a**) and participant 2 (**b**) from post-stimulus 400 ms to 1000 ms. Channels in blue box denote left-LPFC ROI. Yellow-to-red colors denote increased relative power (from 0.1% to 100%), while cyan-to-blue colors denote decreased relative power (from −0.1% to −100%).

In all participants, platinum electrodes were implanted in the left hemisphere of their brain. In the early time window, we did not find any significant relative power difference between impulsive and non-impulsive decisions in the theta, alpha, beta, and gamma oscillations of all participants. However, in the later time window, we found a similar pattern of theta power spectral changes in the two participants, wherein the theta powers of electrodes in the left LPFC were significantly stronger during impulsive decisions than non-impulsive decisions (paired-sample *t* tests, *p* = 0.034 for participant 1, *p* = 0.012 for participant 2, Figs 4 and 5). Participant 1 chose immediate rewards (62%) more frequently in the task than participant 2 (49%). Consequently, participant 1 increased 3.9% of their theta power which was higher than that of participant 2 who increased 0.4% of their theta power. This implied that the more impulsive participant was associated with a higher theta power increase. We found similar patterns of beta power spectral changes in the two participants, wherein the beta powers of electrodes in the LPFC were significantly lower during impulsive decisions than non-impulsive decisions (paired-sample *t* tests, *p* = 0.018 for participant 1, *p* < 0.001 for participant 2, Figs 4 and 5). We also excluded some channels close to premotor area, and performed the paired *t* test again. We still obtained significant results for both participant 1 and participant 2 in theta (paired-sample *t* tests, *p* = 0.017 for participant 1, *p* = 0.026 for participant 2) and beta (paired-sample *t* tests, *p* = 0.001 for participant 1, *p* = 0.039 for participant 2) frequencies, and there was no significant result in other frequencies.

We did not find significant power differences in alpha oscillations during impulsive decisions vs. non-impulsive decisions in the two participants (paired-sample *t* tests, *p* = 0.74 for participant 1, *p* = 0.079 for participant 2). Finally, yet importantly, we did not find significant differences in power spectral changes of gamma (30–80 Hz) (paired-sample *t* tests, *p* = 0.15 for participant 1, *p* = 0.69 for participant 2) and high gamma (80–150 Hz) oscillations (paired-sample *t* tests, *p* = 0.75 for participant 1, *p* = 0.59 for participant 2) between impulsive and non-impulsive decisions. Our results indicate that theta and beta oscillatory activities in the LPFC are important for decision-making during intertemporal choices, while alpha and gamma oscillatory activity during the task was not significantly different between the two decisions in intertemporal choice.

## Discussion

In this study, we used ECoG to investigate how neural oscillations in the LPFC impact intertemporal choice. Our findings revealed that theta and beta oscillations in the LPFC have different functional roles during decision-making. Neural activity of theta oscillation was significantly higher during impulsive decisions, while beta oscillation activity was significantly higher during non-impulsive decisions.

During intertemporal choice, self-control is a crucial factor that influences decision outcome. Studies on impulsivity have indicated that highly impulsive individuals with low self-control tend to choose immediate reward, and individuals with clinical disorders involving self-control, such as gambling and addiction, have abnormal behaviors during intertemporal choice^33–35^. Self-control is linked to prefrontal cortex, and neural activity in the LPFC may be a reliable biomarker for impulsivity and cognitive control^14,21^.

A typical clinical disorder associated with impulsivity, self-control, and prefrontal cortex is attention-deficit/hyperactivity disorder (ADHD). Many studies have shown that patients with ADHD have increased theta and reduced beta oscillations, and the theta-to-beta ratio is a biomarker for ADHD^36–38^. Previous studies and our results suggest that hyperactivity in theta oscillations is associated with increased impulsivity, while stronger beta oscillations are related to cognitive control and non-impulsivity during intertemporal choice.

Our findings suggest a functional dissociation between cortical theta and beta oscillations during the decision-making processes of intertemporal choice. Therefore, decision outcomes may be associated with modulation of the LPFC via neural oscillations at different frequencies. Previous studies have indicated that slow fluctuations in oscillatory power changes are correlated with blood oxygenation level dependent (BOLD) connectivity across distinct brain areas^39^ and modulation of slow-wave and fast-wave oscillations are associated with cortico-subcortical cross-talk^26,27,40,41^. Therefore, impulsive decisions may result from impaired cortical control and hyperactive subcortical activity in reward or motivation systems.

In our study, stronger theta activity during impulsive decisions suggested that the LPFC might be affected by immediate rewards. Previous studies have shown that theta activity on prefrontal cortex is related to novelty processing^42,43^, which recruits the reward system^44,45^. Participant 1 was more impulsive and had a higher relative theta power than participant 2 during impulsive decisions, suggesting that immediate rewards might be more subjectively tempting to the impulsive participant. We believe that the function of theta oscillation in our study is more complex than response conflict. Theta is broadly distributed across the brain particularly during many high-level cognitive processes that include not only response conflict, but also that such as memory encoding and retrieval, working memory retention, and novelty detections^42,46^. In typical cognitive control experiments, conflict at the response level is elicited by priming two competing responses when only one is correct. Reaction times are often used as a dependent measure to quantify the behavioral effects of conflict^47,48^. Therefore, according to previous studies, we examined the RTs of our studies and found that RTs between impulsive and non-impulsive decisions of participant 2 did not have a significant difference (*p* = 0.243), however, theta power changes between impulsive and non-impulsive decisions were significantly different (*p* = 0.012). On the other hand, in typical cognitive control experiments, conflict at the response level is elicited by priming two competing responses when only one is correct, but in our study, there was no correct option for the participants to choose; they arrived at a decision on their own. In many studies, tasks on response conflict were based on typical cognition tasks like the *Stroop*, *Go/NoGo*, and *Flankers* tasks^46,47^. However, intertemporal choice is a decision-making task that requires not only primary cognitive processes, but also advanced and complex decision-making processes, such as value representation, memory retrieval, and self-control^8,9,14,49^.

In contrast, we found that participant 2 had higher changes in beta power than participant 1 during non-impulsive decisions. A stronger beta activity during non-impulsive decisions suggested that the LPFC successfully mediated self-control by inhibiting subcortical activity in the brain reward pathway via top-down neural control. Previous studies have shown that beta power is linked to top–down processing^50^ and impulsivity in intertemporal choice^51^. Similarly, during the Iowa gambling task, in which the participants inhibit options of large but high-risk reward, higher theta-to-beta ratios are associated with lower inhibition ability^40^. In another study, Swann, *et al*.^29^ found that deep brain stimulation of the sub-thalamic nucleus improves behavioral stopping performance and increases beta-band response over the right frontal cortex in patients with Parkinson’s disease as well as healthy controls. Thus, previous studies and our results suggest that beta oscillation in the LPFC is crucial for response inhibition and cognitive control.

A limitation of the present study is the small number of subjects (only two subjects met the inclusion criteria), because ECoG patients are rare and difficult to recruit for the general researching purposes. Thus, future research should enroll more subjects that meet the inclusion criteria to investigate human intracranial cortex activity. Another limitation of the study is that we did not find any significant power spectral changes in gamma (30–80 Hz) and high gamma (80–150 Hz) oscillations between impulsive and non-impulsive decisions. Given the small number of subjects in the current study, we would not draw strong conclusions on the function of gamma oscillation in intertemporal choice. The role of gamma oscillation in decision-making processes during intertemporal choice should be interpreted with caution, and further empirical studies using ECoG or other appropriate techniques are required.

In conclusion, we used ECoG to investigate human intracranial cortex activity and how neural oscillations in the LPFC influence intertemporal choice. We found that neural activities of theta and beta oscillations in the LPFC play different functional roles during the decision-making processes. Our findings suggest that the LPFC may modulate other neural pathways via oscillations with different frequencies. Our study provides empirical evidence for the functional dissociation of cortical theta and beta oscillations in the LPFC during intertemporal choice.
