# Author Correction: Dissociable functional activities of cortical theta and beta oscillations in the lateral prefrontal cortex during intertemporal choice

**DOI:** 10.1038/s41598-019-48220-2

**Published:** 2019-08-15

**Authors:** Dan-Yang Gui, Tao Yu, Zhenhong Hu, Jiaqing Yan, Xiaoli Li

**Affiliations:** 1 Department of Marketing, College of Management, Shenzhen University, Shenzhen, China; 2 State Key Laboratory of Cognitive Neuroscience and Learning & IDG/McGovern Institute for Brain Research, Beijing Normal University, Beijing, China; 3 Beijing Institute of Functional Neurosurgery, Xuanwu Hospital, Capital Medical University, Beijing, China; 4 J. Crayton Pruitt Family Department of Biomedical Engineering, University of Florida, Gainesville, FL 32611-6131 USA; 5 College of Electrical and Control Engineering, North China University of Technology, Beijing, China

Correction to: *Scientific Reports* 10.1038/s41598-018-21150-1, published online 25 July 2018

The Acknowledgements section in this Article is incomplete.

“This research was supported by the National Natural Science Foundation of China (31530031, 81471376), and Scientific Research Foundation for New Teacher of Shenzhen University (2017012). The funding sources had no role in study design, data collection and analysis, decision to publish, or preparation of the manuscript. The authors thank Yu Nan, Wuxia Yang, and Jie Feng for their help with data acquisition. We also thank Liang Wang and Duan Li for data analysis.”

should read:

“This research was supported by the National Natural Science Foundation of China (31530031, 81471376), National Natural Science Foundation of Guangdong Province of China (2018A030310568), and Scientific Research Foundation for New Teacher of Shenzhen University (2017012). The funding sources had no role in study design, data collection and analysis, decision to publish, or preparation of the manuscript. The authors thank Yu Nan, Wuxia Yang, and Jie Feng for their help with data acquisition. We also thank Liang Wang and Duan Li for data analysis.”

